# Impaired function of tendon-derived stem cells in experimental diabetes mellitus rat tendons: implications for cellular mechanism of diabetic tendon disorder

**DOI:** 10.1186/s13287-018-1108-6

**Published:** 2019-01-15

**Authors:** Liu SHI, Ying-juan LI, Guang-chun DAI, Yu-cheng LIN, Gang LI, Chen WANG, Hui CHEN, Yun-feng RUI

**Affiliations:** 1 Department of Orthopaedics, Zhongda Hospital, School of Medicine, Southeast University, No. 87 Ding Jia Qiao, Nanjing, 210009 Jiangsu People’s Republic of China; 2 Orthopaedic Trauma Institute, Southeast University, No. 87 Ding Jia Qiao, Nanjing, 210009 Jiangsu People’s Republic of China; 3 Trauma Center, Zhongda Hospital, School of Medicine, Southeast University, No. 87 Ding Jia Qiao, Nanjing, 210009 Jiangsu People’s Republic of China; 4 Multidisciplinary Team (MDT) for Geriatric Hip Fracture Comprehensive Management, Zhongda Hospital, School of Medicine, Southeast University, No. 87 Ding Jia Qiao, Nanjing, 210009 Jiangsu People’s Republic of China; 5 School of Medicine, Southeast University, No. 87 Ding Jia Qiao, Nanjing, 210009 Jiangsu People’s Republic of China; 6 Department of Geriatrics, Zhongda Hospital, School of Medicine, Southeast University, 87 Ding Jia Qiao, Nanjing, 210009 People’s Republic of China; 7 Department of Orthopaedics and Traumatology, Faculty of Medicine, The Chinese University of Hong Kong, Hong Kong, Hong Kong; 8 Department of Orthopaedics, Xishan People’s Hospital, 588 Guang Rui Road, Wuxi, 214011 Jiangsu People’s Republic of China; 9 China Orthopedic Regenerative Medicine Group, Hangzhou, 310000 Zhejiang People’s Republic of China

**Keywords:** Diabetes mellitus, Tendinopathy pathogenesis, Tendon-derived stem cells, Erroneous differentiation

## Abstract

**Background:**

Patients with diabetes mellitus (DM) often suffered with many musculoskeletal disorders, such as tendon rupture and tendinopathy. However, the understanding of the pathogenesis of these alternations is limited. This study was designed to investigate the role of tendon-derived stem cells (TDSCs) in histopathological alterations of DM tendons.

**Methods:**

Forty-two SD rats were randomly and equally divided into a diabetes group (DG) and control group (CG). DM was induced by streptozotocin (65 mg/kg). The patellar tendons were isolated at weeks 1, 2, and 4 for histological analysis. TDSCs were isolated at week 2 for osteo-chondrogenic differentiation analysis. Mann-Whitney *U* test was used with SPSS. *p* < 0.050 was statistically significant.

**Results:**

Micro-tears of collagen fibers and altered appearance of tendon cells were observed in DG tendons. DG tendons exhibited significantly higher expression of OPN, OCN, SOX9, and Col II and decreased expression of Col I and tenomodulin (TNMD) at week 2. Diabetic TDSCs (dTDSCs) demonstrated significantly decreased proliferation ability and increased osteogenic and chondrogenic differentiation ability. Osteo-chondrogenic markers BMP2, ALP, OPN, OCN, Col II, and SOX9 were also significantly increased while tenogenic markers Col I and TNMD were decreased in dTDSCs.

**Conclusion:**

These results suggested the erroneous differentiation of dTDSCs might account for the structural and non-tenogenic alternations in DM tendons, which provided new cues for the pathogenesis of tendon disorders in DM.

**Electronic supplementary material:**

The online version of this article (10.1186/s13287-018-1108-6) contains supplementary material, which is available to authorized users.

## Background

With the increasing incidence of diabetes mellitus (DM) and the complications [1], such as retinopathy [2], nephropathy [3], and osteoporosis [4], numerous studies were performed to investigate the association between DM and these chronic diseases. However, little attention was focused on the influences of DM on the tendons and ligaments. It is reported that the incidence of tendinopathy in patients with DM was significantly higher than healthy people at the same age [5, 6]. Most of these patients suffered with the chronic pain and limited range of joint motion while some of the others were asymptomatic [7]. Increased thickness of diabetic tendons as well as the disorganized collagen fibers was found with ultrasound and magnetic resonance imaging [8, 9]. The deposition of the calcium at the enthesis of the Achilles tendons was also observed in several DM patients, thus resulted in higher risk of tendon rupture [10, 11]. In experimental DM animal models, the significantly decreased biomechanical properties of the DM tendons were also reported [12–15]. The weaken biomechanical properties might be attributed to the impaired healing process of the tendons in DM tendons with a smaller transverse area and less organized collagen fibers at the tendon-bone interface [13, 14]. In gross view, the tendons in DM subjects showed a yellowish discoloration and much more fragile and atrophic [12, 13]. The arrangement of the collagen fibers in DM tendons was in disorder when compared to the parallel and bundled healthy tendon fibers; the micro-tears were frequently observed as well [16].

In previous studies, we found tendon-derived stem cells (TDSCs) isolated from collagenase-induced tendinopathy rat model exhibited the increased osteo-chondrogenic differentiation potential and decreased tenogenic differentiation potential [17]. Therefore, taken together with the calcium deposition found in the Achilles tendons in DM patients [10], and such non-tenogenic changes in diabetic tendons have not been studied in depth, we raised the hypothesis that diabetic tendons have characteristics of structural and non-tenogenic changes as in classical tendinopathy, and the impaired function of TDSCs might account for these pathological changes, which might provide some implications for the cellular mechanism of the diabetic tendon disorders.

## Methods

### Study design

The 42 SD rats (female, 200–250 g, 8 weeks) were randomly divided into two groups: control group (CG, *n* = 21), DM group (DG, *n* = 21). At weeks 1, 2, and 4 post-induction, 6 rats of each group were sacrificed and the patellar tendons were isolated immediately for histological analysis. At week 2, 3 rats of both groups were sacrificed for TDSC isolation and analysis. All animal experiments were approved by the Animal Experimentation Ethics Committee, School of Medicine, Southeast University (Additional file 1).

### Induction of DM

The method of rat DM model induction with streptozotocin (STZ; Sigma, St. Louis, MO) was well established [12, 18]. Briefly, the rats were induced to DM through intraperitoneal injection of STZ-citrate buffer solution (65 mg/kg) while the CG rats received the citrate buffer only. All rats were fasted for 8 h and subjected to intraperitoneal glucose tolerance test (IPGTT) before and at day 3 post-injection. The sustained phenotype of DM is regarded as the maintained blood glucose (BG) levels equal to or higher than 250 mg/dL continuously. BG levels of all rats were measured every 2 days with a glucometer (ACCU-CHEK® Performa).

### Histological analysis of the patellar tendons

#### General histology and immunohistochemistry

At weeks 1, 2, and 4 post-induction, the patellar tendons were isolated immediately after euthanasia and fixed in 4% paraformaldehyde for 24 h. The tendons were dehydrated through gradient alcohol, embedded in paraffin, and cut longitudinally to 4-μm-thick sections. After deparaffination and hydration, the slides were stained with hematoxylin and eosin (H&E). Immunohistochemistry (IHC) staining was performed as previously described [17, 19]; in brief, the primary antibodies against collagen type I (Col I, Abcam, Cambridge, USA; ab34710; 1:100), tenomodulin (TNMD, Santa Cruz Biotechnology, Santa Cruz, CA; sc-98875; 1:100), osteopontin (OPN, Abcam, Cambridge, USA; ab8448; 1:100), osteocalcin (OCN, Abcam, Cambridge, USA; ab13421; 1:100), SOX9 (Santa Cruz Biotechnology, Santa Cruz, CA; sc-20095; 1:30), and collagen type II (Col II, Abcam, Cambridge, USA; ab34712; 1:100) were used. Goat anti-rabbit (Abcam, Cambridge, USA; ab6721; 1:200) and goat anti-mouse IgG horseradish peroxidase (HRP)-conjugated secondary antibodies (Abcam, Cambridge, USA; ab6789; 1:200) were used according to the primary antibodies then chlorate with 3,3′-diaminobenzidine (DAKO, Glostrup, Denmark). Primary antibody was replaced with blocking solution in the controls. The slides were analyzed under a light microscope (Leica Cambridge, Cambridge, UK), semi-quantitative analysis of IHC staining was performed with Image-Pro Plus software (MediaCybernetics, Bethesda, MD, USA), and the assessors were blind to the sample grouping.

#### Isolation and culture of TDSCs

The procedures of TDSCs isolation and culture have been described previously [17]. Briefly, the middle substance of the patellar tendon was digested with type I collagenase (3 mg/ml, Sigma-Aldrich). The cells were cultured with the complete culture medium which contained low-glucose Dulbecco’s modified Eagle’s medium (Gibco), 10% fetal bovine serum, 100 U/mL penicillin, 100 mg/mL streptomycin, and 2 mM l-glutamine (all from Invitrogen Corporation, Carlsbad, CA). The early passages (P3 to P5) of both healthy TDSCs (hTDSCs) and diabetic TDSCs (dTDSCs) were used for all experiments.

#### Flow cytometry assay

For the flow cytometry assay, both hTDSCs and dTDSCs at P3 were used. 1 × 10^6^ cells of each group were incubated with 1 mg of phycoerythrin-conjugated or fluorescein-isothiocyanate-conjugated monoclonal antibodies (R&D Systems) at 4 °C for 1 h. Anti-CD34 (sc-7324; Santa Cruz Biotechnology, Santa Cruz, CA), anti-CD31 (ab33858; Abcam, Cambridge, UK), anti-CD44 (BD550974; BD Bioscience), anti-CD45 (BD559135; BD Bioscience), and anti-CD90 (BD554898; BD Bioscience) were the antibodies used in this study. Phycoerythrin-conjugated or fluorescein-isothiocyanate-conjugated isotype-matched IgG1 were used as negative controls (IC002P or IC002F; R&D Systems). The stained cells were washed with ice-cold PBS containing 2% BSA before analysis using the LSRFortessa flow cytometer (Becton Dickinson, San Jose, CA). About 1 × 10^4^ events were counted for each sample. The percentage of cells with a positive signal was calculated using the WinMDI Version 2.9 program (The Scripps Research Institute, La Jolla, CA).

#### Colony forming ability assay and cell proliferation assay

For the colony-forming ability assay (CFA) [20], both hTDSCs and dTDSCs at P3 were seeded at the optimal cell density (1000 nucleated cells) on 20 cm^2^ dishes and cultured for 7 to 9 days to form colonies. The number of cell colonies was counted after stained with 0.5% crystal violet (Sigma, St Louis, MO). total number of colonies of both groups were recorded after ignoring the colonies which were less than 2 mm in diameter and were faintly stained.

For the cell proliferation assay, the P3 cells of both groups were seeded on a 96-well plate (4000 cells/well). After incubated for 24, 48, 72, and 96 h, cells were treated with methyl thiazolyl tetrazolium (MTT) solution (0.5 mg/ml) for 4 h then dissolved with dimethyl sulfoxide and shaken for 10 min. The absorbance was measured at 570 nm with a microplate reader.

### Osteo-chondrogenic differentiation potential of hTDSCs and dTDSCs

#### Osteogenic differentiation assay

The methods of TDSC multi-differentiation induction have been described in the previous studies [17, 20]. For the osteogenesis differentiation assay, two kinds of TDSCs were plated at 4 × 10^3^ cells/cm^2^ in 6-well plate and cultured in a complete culture medium until the cells reached 80 to 90% confluence then incubated in both basal medium and osteogenic induction medium (OIM) which was complete culture medium supplemented with 20 mM β-glycerolphosphate, 50 mM ascorbic acid, and 1 nM dexamethasone (all from Sigma-Aldrich) for 7 days. Alkaline phosphatase (ALP) staining and the mRNA expression of ALP, bone morphogenetic protein 2 (BMP2), OPN, and OCN by quantitative real-time reverse transcription-polymerase chain reaction (qRT-PCR) assay were performed. Alizarin red staining (ARS) was done after the basal medium and OIM incubated for 14 days to evaluate the calcium nodule formation.

#### Chondrogenic differentiation assay

The pellet culture system was used for chondrogenic differentiation assay. 8 × 10^5^ cells (P3) were used to form a pellet in a 15-mL conical polypropylene tube by centrifugation at 450*g* for 10 min and cultured in both basal medium and chondrogenic induction medium (CIM) which contained low-glucose Dulbecco’s modified Eagle’s medium (Gibco, Invitrogen Corporation) supplemented with 500 ng/mL BMP-2 (R&D Systems, Inc.), 10 ng/mL transforming growth factor-β3 (R&D), 50 mg/mL ascorbate-2-phosphate, 10^−7^ M dexamethasone, 40 mg/mL proline, 100 mg/mL pyruvate (all from Sigma-Aldrich), and 1:100 diluted ITS + Premix (6.25 mg/mL insulin, 6.25 mg/mL selenous acid, 6.25 mg/mL transferrin, 5.35 mg/ mL linoleic acid, 1.25 mg/mL bovine serum albumin) (Becton Dickinson, Franklin Lakes, NJ) at 37 °C, 5% CO_2_. At day 28, the pellets were either fixed for histological analysis (H&E and Safranin O (SO)) and IHC staining (SOX9 and Col II) or total RNA extraction for mRNA expression as described below.

#### mRNA expression of tenogenic markers

Both hTDSCs and dTDSCs at P3 were seeded at 1 × 10^4^ cells/cm^2^ in a 6-well plate in the complete culture medium at 37 °C, 5% CO_2_. At day 7, the cells were harvested and the mRNA expression of tenogenic markers, including Col I, scleraxis (Scx), and TNMD were examined by qRT-PCR as described below.

#### qRT-PCR assay

qRT-PCR was performed as previously described [21]. Briefly, cells were harvested and homogenized for RNA extraction with RNeasy mini kit (Qiagen GmbH, Hilden, Germany). Then, mRNA was reverse transcribed to cDNA by the First-Strand cDNA kit (Promega, Madison, WI). The condition for PCR was optimized in a conventional PCR machine (GeneAmp 9700; Applied Biosystems, Foster City, CA) for the primers specific for GAPDH, BMP2, OPN, OCN, Col II, and SOX9 as shown in Table 1 at various annealing temperatures (Table 1). Cycling conditions were as follows: denaturation at 95 °C for 10 min, 45 cycles at 95 °C for 20 s, optimal annealing temperature for 30 s, 72 °C for 30 s, and finally at 60–95 °C with a heating rate of 0.1 °C/s. Results were analyzed using ABI StepOne Plus system (Applied Biosystems, Foster City, CA). Expression of the target gene was normalized to GAPDH gene. Relative gene expression was calculated using the 2^−ΔΔCT^ formula.

**Table 1:** Primer sequences and condition for qRT-PCR

Gene	Primer nucleotide sequence	Product size (bp)	Annealing temperature	Accession no
GAPDH	5′-CGGCAAGTTCAACGGCACAG-3′ (forward) 5′-GAAGACGCCAGTAGACTCCACGAC-3′ (reverse)	148	60	NM_017008.4
BMP2	5′-CCCTTTGTATGTGGACTTCAGTGATGTG-3′ (forward) 5′-CTATGGCATGGTTGGTGGAGTTCAG-3′ (reverse)	137	63	NM_017178.1
OPN	5′-CAGTCGATGTCCCTGACGG-3′ (forward) 5′-GTTGCTGTCCTGATCAGAGG-3′ (reverse)	206	60	NM_012881.2
OCN	5′-GGTGCAAAGCCCAGCGACTCT-3′ (forward) 5′-GGAAGCCAATGTGGTCCGCTA-3′ (reverse)	199	60	NM_013414.1
ALP	5′-ACCATTCCCACGTCTTCACATTT-3′ (forward) 5′-AGACATTCTCTCGTTCACCGCC-3′ (reverse)	162	60	NM_013059.1
Col I	5′-CATCGGTGGTACTAAC-3′ (forward) 5′-CTGGATCATATTGCACA-3′ (reverse)	238	55	NM_053356.1
TNMD	5′-CCATGCTGGATGAGAGAGGTTAC-3′ (forward) 5′-CACAGACCCTGCGGCAGTA-3′ (reverse)	72	58	NM_022290.1
Scx	5′-AACACGGCCTTCACTGCGCTG-3′ (forward) 5′-CAGTAGCACGTTGCCCAGGTG-3′ (reverse)	102	58	NM_001130508.1
Col II	5′-GAGTGGAAGAGCGGAGACTACTG-3′ (forward) 5′-CTCCATGTTGCAGAAGACTTTCA-3′ (reverse)	81	55	NM_012929.1
Sox9	5′-CTGAAGGGCTAGGACTGGAC-3′ (forward) 5′-TACTGGTCTGCCAGCTTCCT-3′ (reverse)	140	58	NM_080403.1

#### Statistical analysis

The data was presented in histogram and reported as mean ± standard deviation. Mann-Whitney *U* test was done using SPSS (SPSS, Inc., Chicago, IL; version 16.0). *p* < 0.050 was considered as statistically significant.

## Results

### Induction of DM

The rats treated with STZ solution demonstrated that a sustained phenotype of DM with mean BG value in DG subjects was sustained higher than 250 mg/dL (Table 2). The mean area under the curve (AUC) in IPGTT of DG subjects also significantly increased when compared with the controls 3 days post-STZ injection (22,530 ± 2190 and 8956 ± 1126, respectively) (**p = 0.002), which provided a quantitative index of the severity of hyperglycemia in DG subjects (Table 2), thus indicating the sustained hyperglycemia of DG rats after STZ injected.

**Table 2:** Results of BG value and IPGTT of both CG and DG rats

	BG level (mg/dL)	Mean AUC
CG	76.3 ± 1.5	8643.6 ± 395.2
DG	344.7 ± 13.9*	22,361.2 ± 1009.8*

*p < 0.01 compared with CG

### Macroscopy observation of pathological changes

#### Histopathology of the patellar tendons in DG rats

**Fig. 1:**
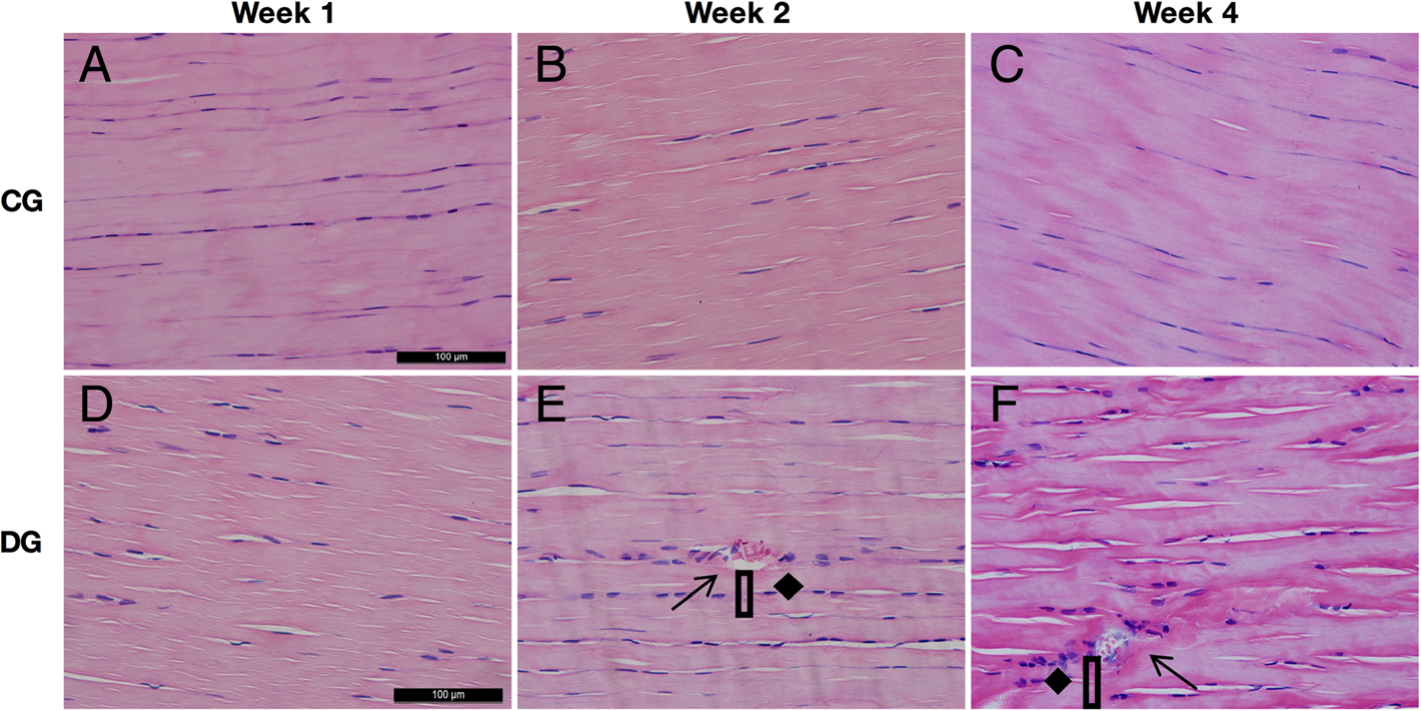
Representative images of H&E staining of both CG and DG patellar tendons at weeks 1, 2, and 4 post-STZ induction. 1A–1C: All of the CG tendons at different time points showed the tight, parallel arrangement of collagen fibers distributed at the same orientation with slight waves. The bundles of collagen fiber were about the same size as well as the tendon cells. 1D–1F: Alternations of collagen fibers arrangement, micro-tears of collagen fibers (arrow), the rounded tendon cells (diamond), and even some red blood cells and blood vessels (rectangle) were observed in diabetic tendons. *n* = 6 of each group. Scale bar = 100 μm

The patellar tendons in DG subjects exhibited disorder arrangement of collagen fibers when the controls demonstrated the tight, parallel arrangement with slight waves (Fig. 1 (1A–1F)). What is more, the micro-tears were frequently observed in diabetic tendons at weeks 2 and 4 post-induction (Fig. 1 (1E, 1E), arrow). The red blood cells and small blood vessels (Fig. 1 (1E, 1E), rectangle) and the rounding changed tendon cells surrounding the tear sites were observed in the diabetic tendons at weeks 2 and 4 post-induction (Fig. 1 (1E, 1E), diamond) while healthy tendon cells in slender shape were well distributed within the longitudinally arranged collagen fibers. These results suggest the DG patellar tendons exhibited the pathological alternations at the early stage of STZ-induced DM rats.

### IHC staining of the patellar tendons

#### Expression of osteo-chondrogenic differentiation markers

**Fig. 2:**
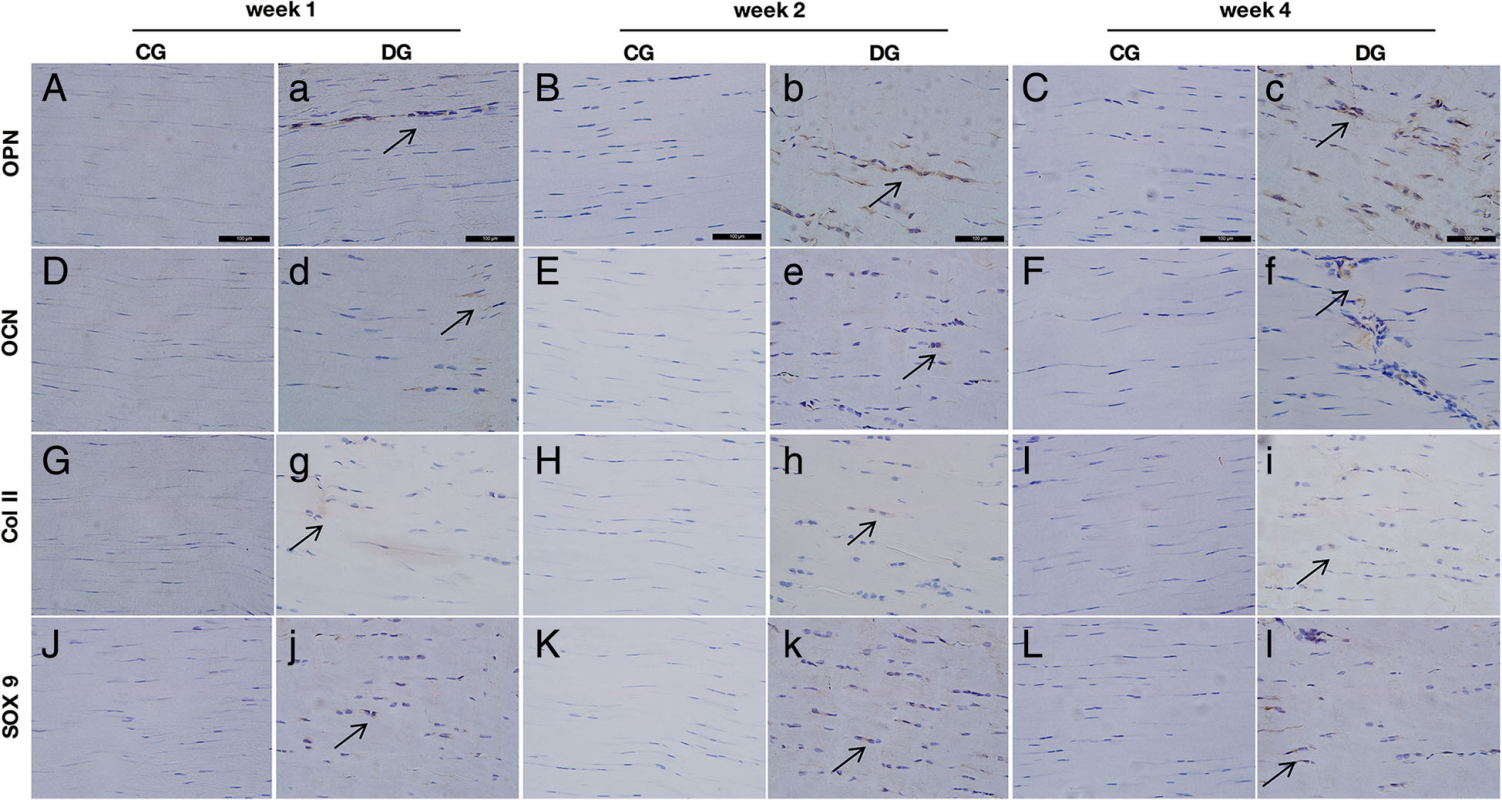
Representative images of IHC staining of osteo-chondrogenesis markers, OPN, OCN, Col II, and SOX9 in CG and DG tendons at weeks 1, 2, and 4 post-STZ induction. The osteogenic markers OPN and OCN were both positively expressed in DG tendons (2a–2f, arrows) at weeks 1, 2, and 4 while the controls were negatively expressed (2A–2F). The chondrogenesis markers Col II and SOX9 were also positively expressed in DG tendons (2g–2 l, arrows) at weeks 1, 2, and 4 while the controls were negatively expressed (2G–2L). *n* = 6 of each group. Scale bar = 100 μm

The expression of osteogenic differentiation markers, including OPN and OCN, were positively stained in the DG tendons while the CG group showed to be negative (Fig. 2 (2A–2H, 2a–2h)). OPN and OCN were mainly expressed in the cytoplasm of diabetic tendon cells, especially surrounding these rounded tendon cells (Fig. 2 (2a–2f), arrow). The chondrogenic differentiation markers, including SOX9 and Col II, were also positively expressed in the diabetic tendon while the CG group showed the negatively stained (Fig. 2 (2G–2L, 2g–2l)). SOX9 was found positively stained in both the nucleus and the cytoplasm of these cells while Col II mainly expressed in the extracellular matrix of tendon cells (Fig. 2 (2g–2l), arrow). These significantly increased osteo-chondrogenic differentiation markers in DG tendon further illustrated the pathological changes in STZ-induced DM rats.

#### Expression of tenogenic markers

**Fig. 3:**
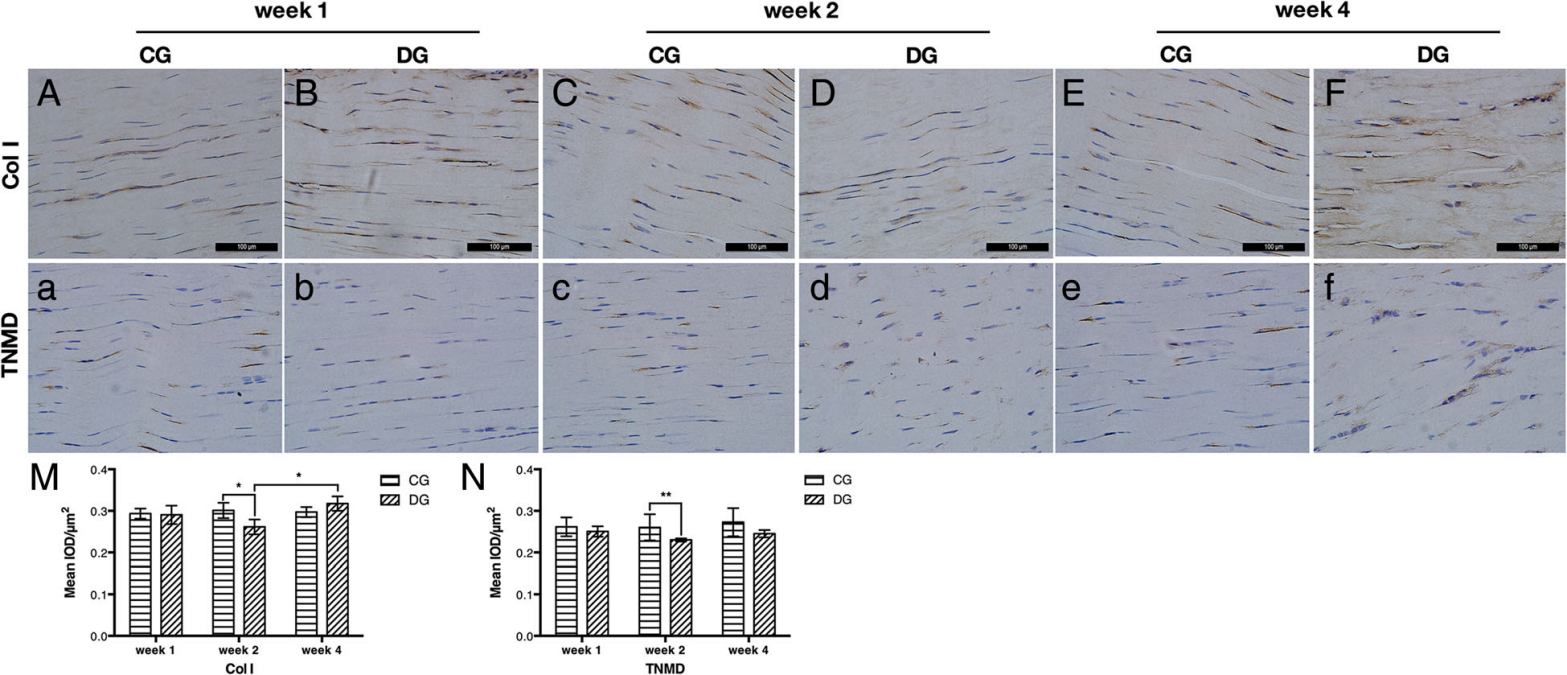
Representative images of IHC staining of tenogenic markers, Col I and TNMD, in CG and DG tendons at weeks 1, 2, and 4 post-STZ induction. The tenogenic markers Col I (3A–3F) and TNMD (3a–3f) were both positively expressed in both groups at weeks 1, 2, and 4, and the semi-quantitative analysis showed the significantly decreased mean IOD/μm^2^ of Col I (3M) and TNMD (3N) in DG tendons at week 2. The expression of Col I in DG tendons turned to be upregulated at week 4 than that of week 2 (3M). *n* = 6 of each group. *p < 0.050, **p < 0.010. Scale bar = 100 μm

All the patellar tendons of both DC and CG subjects showed positive expression of Col I and TNMD (Fig. 3 (3A–3F, 3a–3f)). The semi-quantitative analysis of IHC staining showed the significantly decreased mean integrated optical density (IOD)/μm^2^ of Col I and TNMD in the patellar tendon of the DG at week 2 post-induction when compared with the controls (Fig. 3 (3M, 3N), *p = 0.010 and **p = 0.004, respectively). Interestingly, the expression of Col I in diabetic tendons was remarkably increased at week 4 than that of week 2 (Fig. 3 (3M), *p = 0.010). These histological and IHC results demonstrated the tendons in STZ-induced DG rats exhibited the characteristics of structural and non-tenogenic alternations as in classical tendinopathy.

#### Immunophenotypes and proliferative capacity of TDSCs

**Fig. 4:**
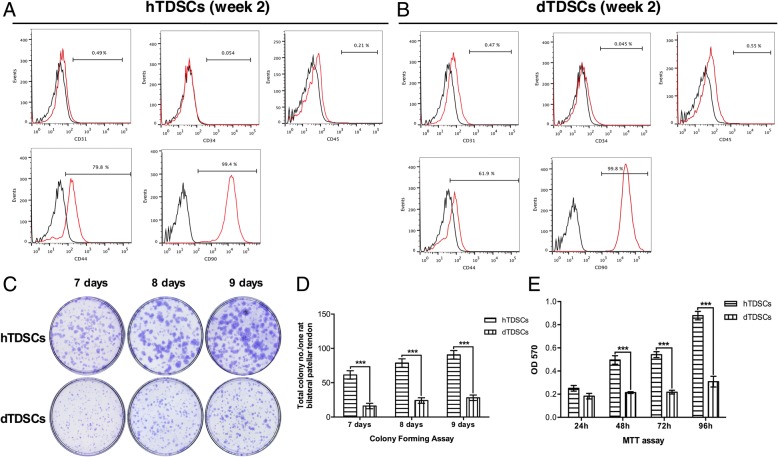
4A, 4B: The histogram showed the surface expression of endothelial stem cell marker (CD31), hematopoietic lineage markers (CD34 and CD45), and mesenchymal stem cell markers (CD90 and CD44) on hTDSCs and dTDSCs. 4C, 4D: The CFA showed the decreased colony-forming capacity of dTDSCs than hTDSCs. *N* = 3 of each group. 4E: The MTT assay also showed the significantly decreased proliferation ability of dTDSCs. *N* = 6 of each group. ***p < 0.001

TDSCs were isolated from both CG and DG rat patellar tendons after 2 weeks post-STZ induction in this study. Both hTDSCs and dTDSCs were positive for mesenchymal stem cell markers, including CD90 and CD44, but negative for hematopoietic lineage markers, including CD34 and CD45, and endothelial cell markers, including CD31. The surface expression of CD44 was remarkably reduced in dTDSCs (61.9%) compared to hTDSCs (79.8%) (Fig. 4 (4A, 4B). Less TDSC colonies were found in DG group at 7, 8, and 9 days when compared with matched controls (Fig. 4 (4C, 4D), all ***p < 0.001). In addition, the results of MTT reduction assay also suggested the significantly decreased proliferative ability of dTDSCs at 48, 72, and 96 h (Fig. 4 (4E), all ***p < 0.001).

#### Osteogenic differentiation potential

**Fig. 5:**
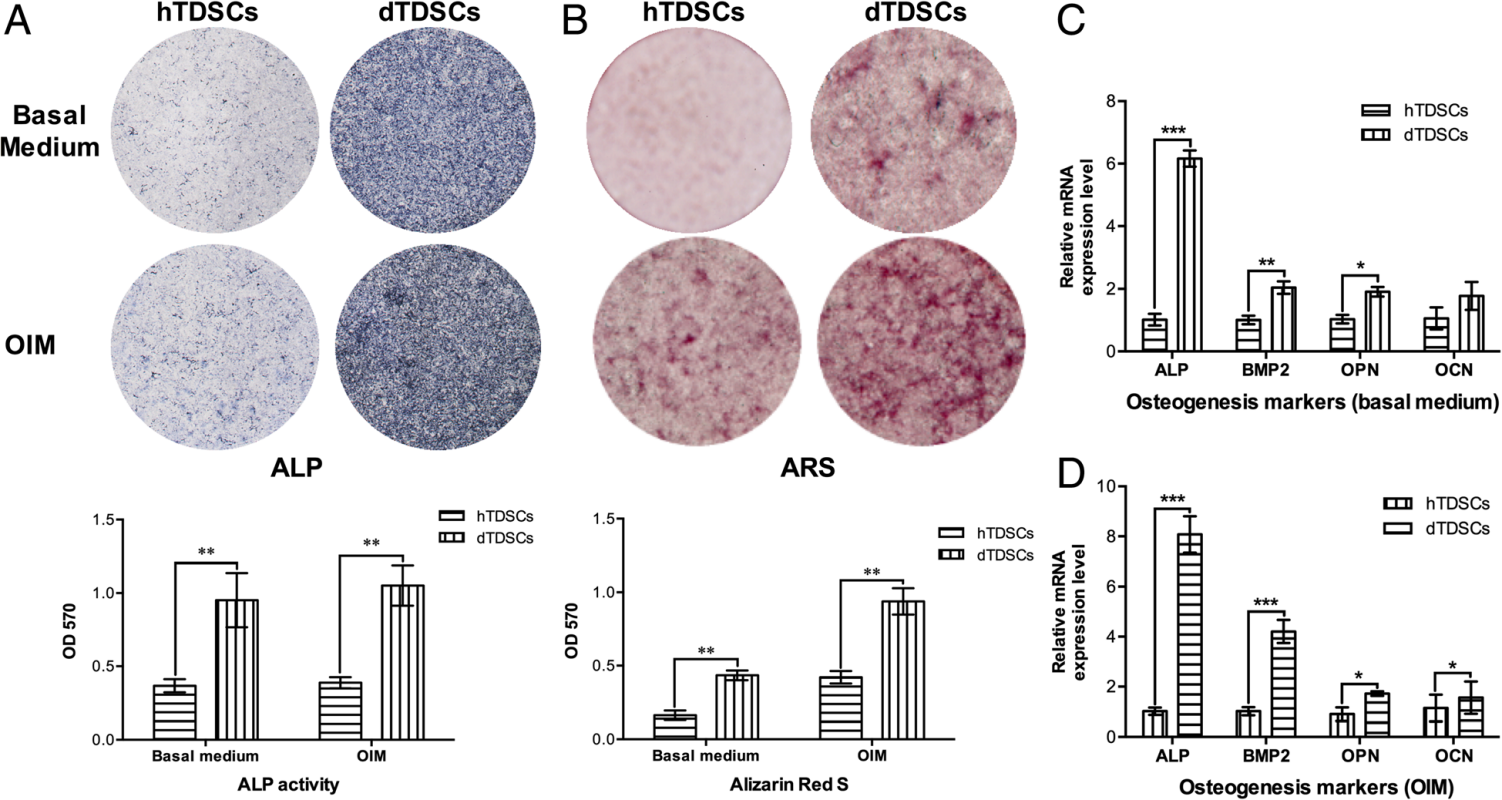
The result of osteogenic differentiation assay. 5A, 5B: The ALP activity was evaluated at day 7. The ARS activity was examined at day 14. 5C, 5D: The expression of osteogenesis-related marker genes was measured by qRT-PCR assays at day 7 under both basal medium and OIM incubation. *N* = 3 of each group. *p < 0.05, **p < 0.01, ***p < 0.001

In the osteogenic differentiation assay, the dTDSCs showed significantly higher ALP activity than the hTDSCs at day 7 under both basal medium and OIM incubation (Fig. 5 (5A), **p = 0.002 and **p = 0.002, respectively). Meanwhile, more ARS-positive calcium nodules were found in dTDSCs at day 14 post-basal medium and OIM incubation. The quantification analysis also showed the dTDSCs exhibited a significantly higher intensity of the calcium-bound ARS signal at day 14 in both basal and OIM when compared (Fig. 5 (5A), **p = 0.004 and **p = 0.001, respectively). What is more, the expression of ALP (***p < 0.001), BMP2 (**p < 0.001), OPN (*p = 0.030), and OCN (*p = 0.020) of dTDSCs also remarkably upregulated at mRNA level when compared with the hTDSCs under OIM incubation for 7 days (Fig. 5 (5D)). The mRNA expression of ALP (***p < 0.001), BMP2 (**p = 0.004), and OPN (*p = 0.022) of dTDSCs were significantly increased as well under basal medium incubation for 7 days (Fig. 5 (5C)). Taken together, these findings indicated that the osteogenic differentiation potential of TDSCs in STZ-induced DM rats was upregulated in vitro.

#### Chondrogenic differentiation potential

**Fig. 6:**
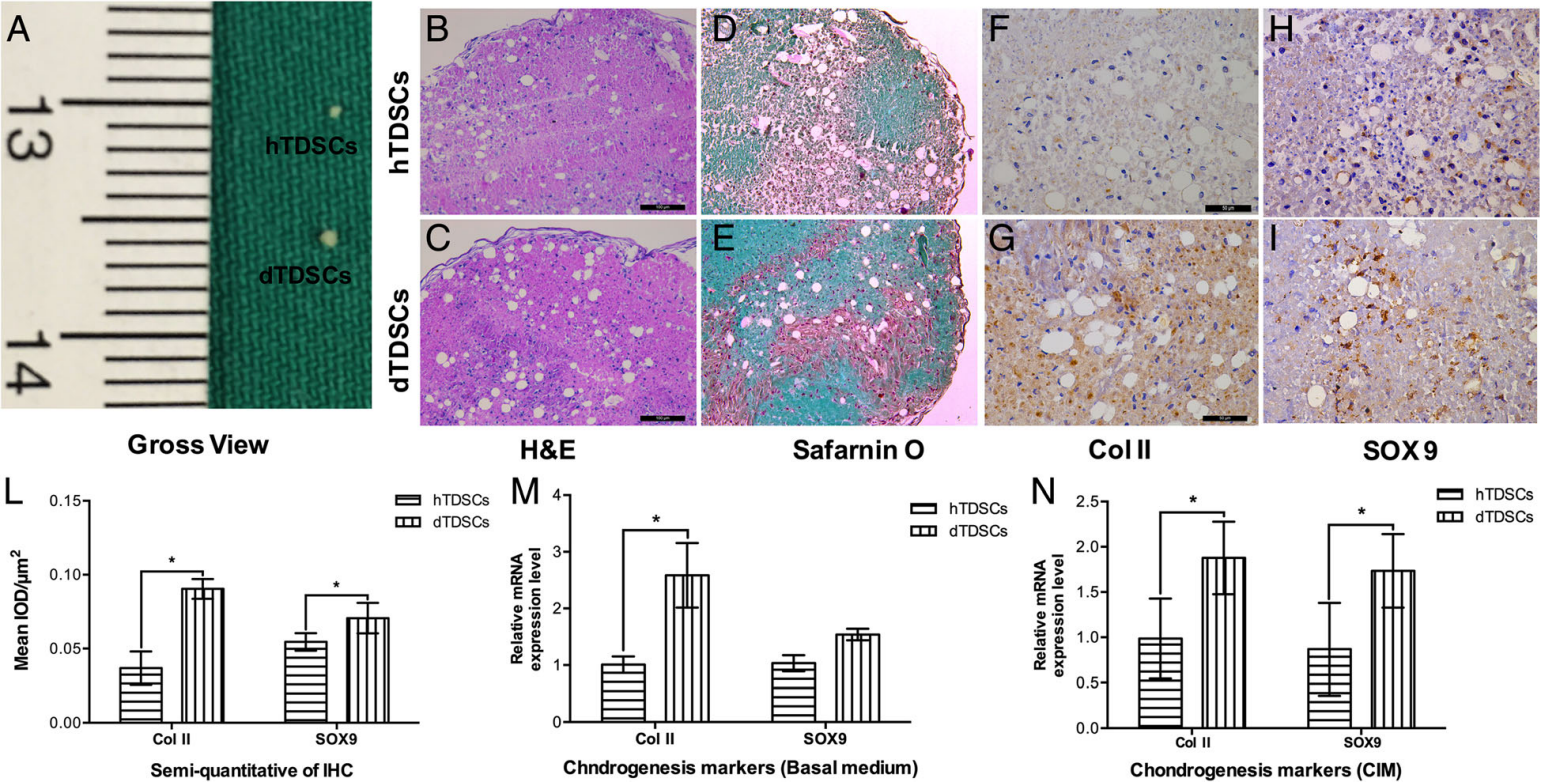
The results of chondrogenesis differentiation assay. 6A: The gross view of the pellets of both hTDSCs and dTDSCs. The histological staining showed more chondrocyte-like cells with H&E staining (6B, 6C) and much higher proteoglycan deposition with Safranin O staining (6D, 6E) in dTDSCs formed pellets after CIM induced for 28 days. Scale bar = 100 μm. 6F–6I, 6L: The expression of Col II and SOX9 were also increased in dTDSCs than the controls with IHC staining. Scale bar = 50 μm. 6M, 6N: The expression of chondrogenesis-related marker genes was measured by qRT-PCR assays at day 14 under both basal medium and CIM incubation. *N* = 3 of each group. *p < 0.05

In the chondrogenic differentiation assay, both hTDSCs and dTDSCs formed pellets at day 28 after chondrogenic induction in a 3D pellet culture system while these induced in basal medium failed to form pellets. The pellet of dTDSCs was much larger than that of hTDSCs in gross view (Fig. 6 (6A)). More chondrocyte-like cells (Fig. 6 (6B, 6C)) and much higher expression of proteoglycan deposition were detected (Fig. 6 (6D, 6E)) in the dTDSCs group than the hTDSCs group. What is more, the expression of Col II and SOX9 was also significantly increased in dTDSCs with IHC staining (Fig. 6 (6F–6I, 6L), *p = 0.050 and *p = 0.050, respectively). The qRT-PCR data also demonstrated that the expression of both Col II and SOX9 in dTDSCs was significantly upregulated at mRNA level under CIM induction for 7 days (Fig. 6 (6N), *p = 0.016 and *p = 0.030, respectively), and the expression of Col II was also increased in dTDSCs under basal medium incubation (Fig. 6 (6N), *p = 0.011). All these findings suggested the dTDSCs possessed higher chondrogenic differentiation potential than hTDSCs.

#### Expression of tenogenic markers

**Fig. 7:**
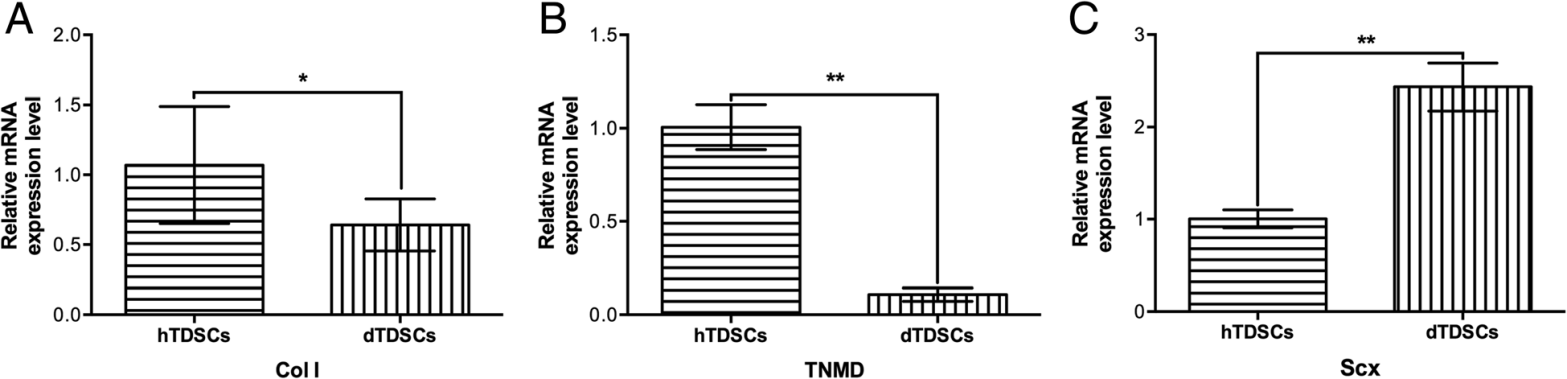
The qRT-PCR results of tenogenic markers. 7A, 7B: The significantly decreased Col I and TNMD expression in dTDSCs than the controls. 7C: The expression of Scx was significantly upregulated in dTDSCs than the hTDSCs. *N* = 3 of each group. *p < 0.050, **p < 0.010

The tenogenic ability of hTDSCs and dTDSCs was compared by investigating the mRNA expression of tenogenic markers, including Col I, TNMD, and scleraxis (Scx). There was a significantly lower mRNA expression of both Col I (Fig. 7 (7A), *p = 0.041) and TNMD (Fig. 7 (6B), **p = 0.002) in dTDSCs when compared with the hTDSCs in basal medium. Interestingly, the expression of Scx was observed to be significantly increased in dTDSCs when compared with the hTDSCs in basal medium (Fig. 7 (6C), **p = 0.002).

## Discussion

The underlying mechanism of the pathological changes in diabetic tendons still remained unclear [6, 22]. In the present study, we investigated the non-tenogenic histopathological alternations in the patellar tendon of experimental type I DM rats and the properties of the TDSCs in the diabetic tendon.

STZ could enter the pancreatic islet B cells via a glucose transporter and cause rapid destruction of B cells and induced subjects to type I DM [18]. Therefore, it is widely used to establish the DM animal model in many studies [12, 18]. After injected STZ solution for 3 days, the results of IPGTT indicated the diabetes phenotype of DG rats in our study and the sustained higher BG level also showed the DM model was successfully established.

In the current study, the histological analysis showed the characteristic tendinopathy alternations in diabetic tendons including the unparalleled arrangement of collagen fibers, disorganized tendon cells in the extracellular matrix, and the rounding changed tendon cells (Additional file 2). The micro-tears, red blood cells, and blood vessels were also discovered in DG tendons while the vessels could not be found in CG tendons. The increased number of vessels in tendons of both DM patients and animal models was also reported [14]. They discovered that the cross-sectional area of these proliferous vessels was also larger, and the vascular endothelial growth factor (VEGF) was also significantly increased in DM tendons [14]. Scientific evidences showed the good blood supply and innervation were essential for the collagen synthesis formation at the injured sites of tendons [23]. Therefore, the increased angiogenesis and upregulated VEGF expression also indicated the formation of chronic tendinopathy in DM subjects. In addition, the deposition of calcium in the Achilles tendon of patients with DM was reported in the clinical study [10]. The fibrocartilage metaplasia was also significantly increased in DM tendons accomplished with stenosing flexor tenosynovitis when compared with non-diabetic subjects [24]. Local hypoxia which might be induced by the micro-tears of collagen fibers and disturbance of micro-circulation were the factors that might contribute to the calcification in DM tendons [25]. These alternations implied the degeneration of DM tendons. These degenerative features might lead to the decreased biomechanical properties of DM tendons when compared to healthy subjects and further resulted in the chronic pain and tendon rupture in some DM patients [11–13].

Furthermore, the positive expression of osteo-chondrogenic markers, including OPN, OCN, Col II, and SOX9, was observed in diabetic tendons while the CG tendons showed the negative expression, thus indicating some of the tendon cells in DM tendons exhibited higher non-tenogenic differentiation potential in vivo. Moreover, the expression tenogenic markers (Col I and TNMD) was decreased in diabetic tendons at week 2 when compared to matched controls. In our previous study, we found the proliferation ability of TDSCs was significantly decreased after treated with high glucose (15 mM (270 mg/dl) and 25 mM (450 mg/dl)) for 1 and 3 days, and the tendon-related markers were also downregulated in vitro [26]. In DM tendon healing models, the proliferation potential of the DM rat tendon cells was also declined in vitro, and the synthesis of Col I and type III collagen of DM tendon was decreased after injury as well [15]. Taken together, these results suggested that the patellar tendon in STZ-induced DM rats might suffer a transitorily decrease of the collagen production at the early stage of the experimental type I DM subjects.

TDSCs exhibit universal stem cell characteristics, including clonogenicity, proliferative capacity, multi-differentiation potential, and mesenchymal stem cell marker expression [20]. Erroneous differentiation of TDSCs towards osteogenesis and chondrogenesis was reported to be a pathogeny for chronic tendinopathy [17]. Hence, we speculate the fate of TDSCs in DM rats was also changed and the erroneous differentiation of these stem cells might lead to the chondrocyte-like change of the tendon cells and the calcium deposition in these tendons.

According to the histological and IHC results, the patellar tendons showed the declining collagen production than the controls after STZ injection for 2 weeks; thus, we isolated the TDSCs at this time point in order to dig out the possible changes at cellular level. Both hTDSCs and dTDSCs positively express mesenchymal stem markers including CD90 and CD44, and the expression of CD44 in dTDSCs was decreased than hTDSCs, as a cell-surface glycoprotein, CD44 participates in cell growth, survival, differentiation, and motility processes [27]. It is reported that knockdown of CD44 could lead to the promoted differentiation of cancer stem cells [28]. Thus, the reduction of CD44 expression in dTDSCs might be related to the altered multi-differentiation potential.

Our results showed the significantly decreased proliferation capacity of dTDSCs when compared to the hTDSCs in vitro; this was consistent with the previous finding that high glucose could suppress the proliferation ability of TDSCs in vitro [26]. What is more, the upregulated ALP activity, calcium deposition, and higher proteoglycan deposition in dTDSCs formed pellets and increased mRNA expression of osteogenesis and chondrogenesis markers, suggesting the significantly enhanced osteo-chondrogenesis differentiation potential of dTDSCs than that of hTDSCs. In addition, we also observed the downregulated tenogenic markers Col I and TNMD expression at mRNA level. Previously, we also found the TDSCs treated with high glucose for 24 and 28 h showed significantly decreased TNMD and Col I expression in vitro [26]. The spontaneous tenogenic differentiation was also reported to be a specific feature of TDSCs [29], manifested the important role of TDSCs in tendon metabolism and repair. However, the dTDSCs showed the decreased tenogenic differentiation potential with significantly increased osteo-chondrogenic differentiation potential than the hTDSCs at week 2 post-STZ induction; this might account for the decreased collagen production and increased non-tenogenic alternations in DM tendons.

Interestingly, the expression of Scx in dTDSCs was significantly upregulated simultaneously. As a transcriptional regulator, Scx is abundantly expressed in embryonic and adult tendons and reported to be re-expressed after tendon injury [30]. The increased expression of Scx might be due to the chronic tendinopathy in DM tendons; the micro-tears of tendon fibers in DM tendons and decreased collagen expression in the extracellular matrix might lead to the upregulated expression of Scx in transcription level [31]. Taken together, the upregulated osteo-chondrogenesis and downregulated tenogenic differentiation potential of TDSCs in DM tendons might be contributed to the increased expression of OPN, OCN, Col II, and SOX9 and decreased expression of Col I and TNMD in diabetes tendons with IHC staining and further resulted in the decreased mechanical properties and degenerative changes, even characteristic tendinopathy alternations in DM tendons.

Nevertheless, the limitations in this study were that the stem cells which we isolated from both control and DM rats could be exogenous or endogenous since the specific marker for TDSCs was not well established, and the composition of the TDSCs was also up to the pathological status of the rats. The other was the direct influence of STZ on tendons, and TDSCs are not studied in depth. It is reported the chronic effect of STZ on tendon mechanical properties in DM animals was due to the high glucose level in vivo after STZ injection [12, 32]. Lehner et al. reported that tendons in non-diabetic human and rats contained a population of pancreas beta cell-like cells which also producing insulin and glucose transporter 2 (GLUT2) [33]. GLUT2 is a key protein which could also transport STZ into the cells [34]; thus, they thought STZ could reduce tendon mechanical strength by affecting tendon cells after a single IP injection of STZ for 5 days. However, they treated the tendons with STZ and showed no apparent structural alterations in vitro. What is more, we also treated the TDSCs with STZ; the osteogenic differentiation potential of TDSCs was not affected in vitro during OIM induction. The expression of GLUT2 was not increased after STZ is added. Therefore, we speculate the primary reason for the activation of these GLUT2-expressing cells in tendon tissue might be due to the systematic increased high glucose level in vivo.

In our previous study, we investigated the direct effects of high glucose on TDSCs in vitro and discovered the expression of tenogenic markers including Tnmd and Col I in TDSCs treated with high glucose were significantly decreased. Actually, we have not investigated the effects of high glucose on the osteo-chondrogenic differentiation of TDSCs.

It is reported that various signaling pathways, such as WNT/β-catenin and TGF-β, were activated in the osteogenic differentiation of tendon-derived cells and TDSCs [35, 36]. The WNT/β-catenin signaling pathway in MSCs osteogenic differentiation was well studied [37]. Therefore, the enhanced osteogenic differentiation in dTDSCs might be attributed to the triggering of WNT/β-catenin; the molecular mechanism of these observed erroneous differentiation of TDSCs and dTDSCs in our previous and present studies needs further investigation.

## Conclusions

**Fig. 8:**
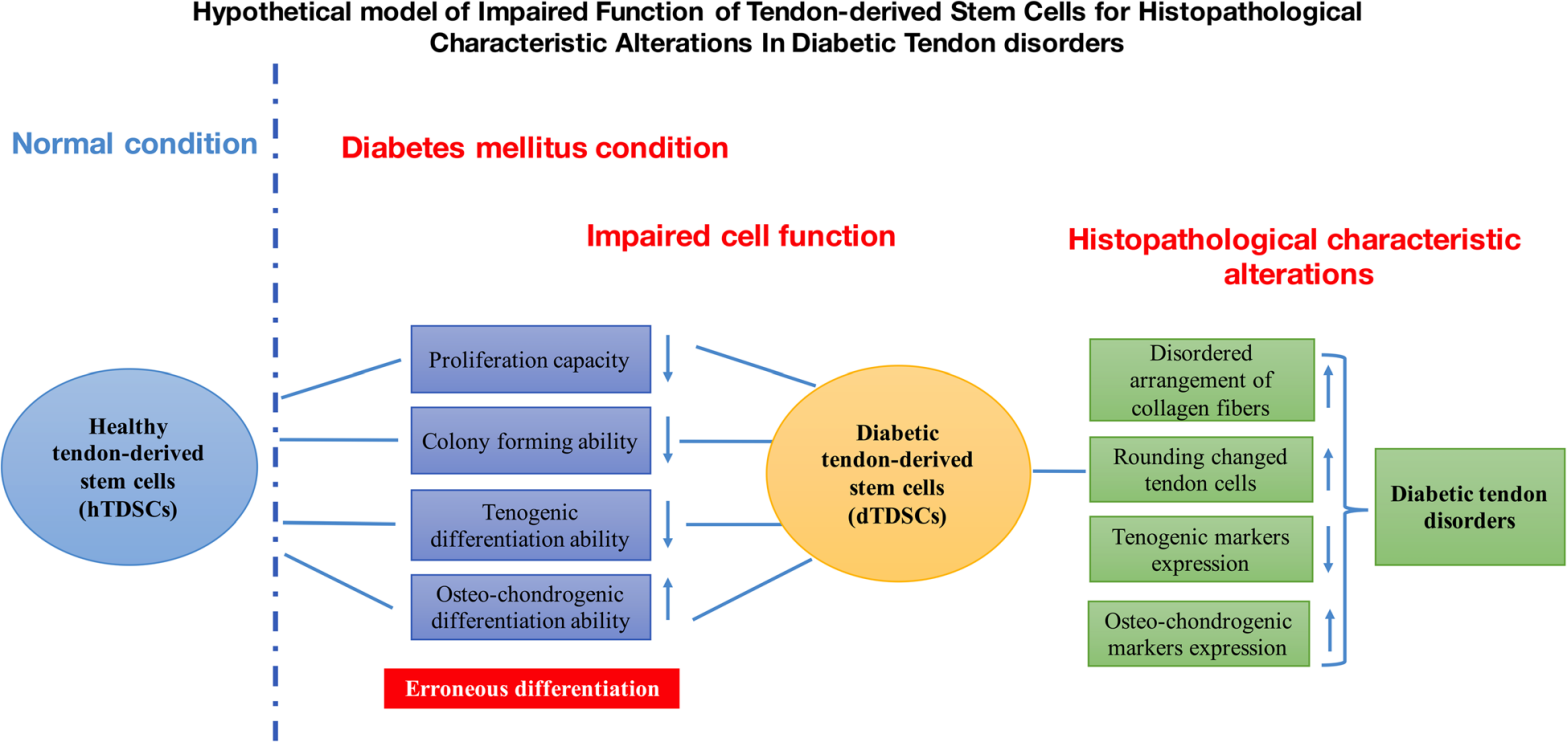
The hypothetical model of impaired function of tendon-derived stem cells for histopathological characteristic alterations of diabetic tendon disorders

In summary, we could also conclude that the dTDSCs which isolated from STZ-induced DM rats showed the significantly decreased proliferation capacity and colony-forming ability than the normal healthy TDSCs, and the erroneous differentiation of dTDSCs with higher osteo-chondrogenic and lower tenogenic differentiation abilities might contribute to the non-tenogenic alternations of tendons in STZ-induced DM rats, further impair its self-managing ability, and develop into histopathological characteristic alterations of diabetic tendon disorders (Fig. 8). Further studies are also needed to explore the potential molecular mechanism of the pathological alternations in DM tendons.

## Additional files

Additional file 1: Ethics approval for animal experimentation. This is the file of the ethics approval for animal experimentation in School of Medicine, Southeast University. (PDF 362 kb)

Additional file 2: Polarizing microscopy view. The typical collagen birefringence was lost in DG tendons when compared with the CG tendons at week 4 under polarizing microscope. (PNG 266 kb)

## Abbreviations

ALP: Alkaline phosphatase
ARS: Alizarin red staining
AUC: Area under the curve
BG: Blood glucose
BMP2: Bone morphogenetic protein 2
CFA: Colony-forming ability assay
CIM: Chondrogenic induction medium
Col I: Collagen type I
Col II: Collagen type II
DM: Diabetes mellitus
dTDSCs: Diabetic TDSCs
GLUT2: Glucose transporter 2
H&E: Hematoxylin and eosin
HRP: Horseradish peroxidase
hTDSCs: Healthy TDSCs
IHC: Immunohistochemistry
IOD: Integrated optical density
IPGTT: Intraperitoneal glucose tolerance test
MTT: Methyl thiazolyl tetrazolium
OCN: Osteocalcin
OIM: Osteogenic induction medium
OPN: Osteopontin
qRT-PCR: Quantitative real-time reverse transcription-polymerase chain reaction
Scx: Scleraxis
SO: Safranin O
STZ: Streptozotocin
TDSCs: Tendon-derived stem cells
TNMD: Tenomodulin
VEGF: Vascular endothelial growth factor
