# Supplementary material for: Impaired function of tendon-derived stem cells in experimental diabetes mellitus rat tendons: implications for cellular mechanism of diabetic tendon disorder
Source: Stem Cell Res Ther. 2019 Jan 15;10:27. doi: 10.1186/s13287-018-1108-6 (PMC6332703; doi:10.1186/s13287-018-1108-6)
Supplement: Supplementary file 1 — Ethics approval for animal experimentation. This is the file of the ethics approval for animal experimentation in School of Medicine, Southeast University. (PDF 362 kb) [file 13287_2018_1108_MOESM1_ESM.pdf]

东南大学附属中大医院临床研究伦理委员会

IEC for Clinical Research of Zhongda Hospital, Affiliated to Southeast University

科研项目伦理审查批件

Approval Letter for Research Project

批件号：2016ZDSYLL038.0

<table>
<tr><td>审查日期</td><td>2016.05.26</td><td>审查地点</td><td>中大医院第一会议室</td></tr>
<tr><td>研究机构</td><td colspan="3">东南大学附属中大医院</td></tr>
<tr><td>项目名称</td><td colspan="3">糖尿病肌腱病变的病理新机制：肌腱干细胞错误分化作用及其调控机制的研究</td></tr>
<tr><td>申办者</td><td colspan="3">不适用</td></tr>
<tr><td>研究科室</td><td>骨科</td><td>项目负责人姓名、职称</td><td>芮云峰、副主任医师</td></tr>
<tr><td>项目类型</td><td colspan="3">■实验性研究
□观察性研究：□回顾性分析　　□前瞻性研究</td></tr>
<tr><td>药物/器械注册分类</td><td colspan="3">不适用</td></tr>
<tr><td>审查类别</td><td>纵向课题立项审查</td><td>审查方式</td><td>■会议审查　　□快速审查</td></tr>
<tr><td>审查文件</td><td colspan="3">1. 科研立项项目伦理审查申请表
2. 国家自然科学基金申请书（2015）
3. 知情同意书（02 版，2016. 05. 13）
4. 科研项目批文
5. 课题项目负责人履历表</td></tr>
<tr><td>审查意见</td><td colspan="3">根据 WMA《赫尔辛基宣言》、CIOMS《人体生物医学研究国际道德指南》、卫生部《涉及人的生物医学研究伦理审查办法（试行（2007））》、CFDA《药物临床试验质量管理规范（2003）》、《医疗器械临床试验规定（2004）》、《药物临床试验伦理审查工作指导原则（2010）》，经本伦理委员会审查，同意按所批准的临床研究方案、知情同意书、招募材料开展本项研究。
请遵循 GCP 原则，遵循伦理委员会批准的方案开展临床研究，保护受试者的健康与权利。
伦理委员会对该批准项目进行跟踪审查直至研究结束。具体要求为：
1.对已批准的项目设计书、知情同意书、招募材料的任何修改及项目负责人的变更等，提交修正案申请，在获得本院伦理委员会批准/肯定答复前不得实施方案的任何偏离和修改。
2. 发生严重不良事件及影响研究风险受益比的非预期不良事件，提交严重不良事件报告。
3.暂停/终止研究，提交暂停/终止研究报告。
4.研究纳入了不符合纳入标准或符合排除标准的受试者，符合中止研究规定而未让受试者退出研究，给予错误治疗或剂量，给予方案禁止的合并用药等没有遵从方案开展研究的情况，或可能对受试者权益/健康以及研究的科学性造成不良影响等违背 GCP 原则的情况，提交违背方案报告。
5. 完成临床研究，提交结题报告。
该批件有效期为 3 年，在有效期到期前一个月（2019 年 05 月 07 日前）向伦理委员会提出延长期限申请。
*审查会议的委员签到表附后。</td></tr>
<tr><td>主任委员签名/日期</td><td colspan="3">[illegible]　　2016.6.8</td></tr>
<tr><td colspan="4">东南大学附属中大医院临床研究伦理委员会（盖章）
东南大学附属中大医院临床研究伦理委员会</td></tr>
<tr><td colspan="4">地址:南京鼓楼区丁家桥 87 号　邮编：210009　联系人：王慧萍　联系电话：025-83272015</td></tr>
</table>
