# Supplementary figures and images for: Impaired function of tendon-derived stem cells in experimental diabetes mellitus rat tendons: implications for cellular mechanism of diabetic tendon disorder
Source: Stem Cell Res Ther. 2019 Jan 15;10:27. doi: 10.1186/s13287-018-1108-6 (PMC6332703; doi:10.1186/s13287-018-1108-6)

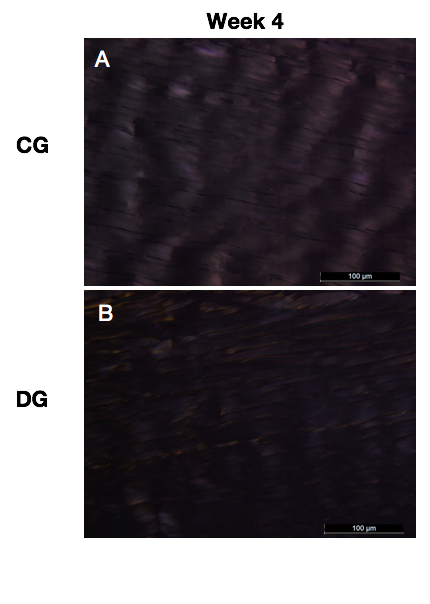

Supplement: Supplementary file 2 — Polarizing microscopy view. The typical collagen birefringence was lost in DG tendons when compared with the CG tendons at week 4 under polarizing microscope. (PNG 266 kb) [file 13287_2018_1108_MOESM2_ESM.png]
